# Clinical, ultrasonographic, and post-mortem diagnosis of respiratory disease in lambs: hematological and biochemical characterization by severity grade

**DOI:** 10.3389/fvets.2026.1807749

**Published:** 2026-03-17

**Authors:** Alejandro Sánchez-Fernández, Juan Carlos Gardón, Marta González, Begoña Álvarez, Joel Bueso-Ródenas

**Affiliations:** 1 Doctoral School, Catholic University of Valencia San Vicente Mártir, Valencia, Spain; 2 Department of Medicine and Animal Surgery, Faculty of Veterinary Medicine and Experimental Sciences, Catholic University of Valencia San Vicente Mártir, Valencia, Spain; 3 Department of Animal Production and Public Health, Faculty of Veterinary Medicine and Experimental Sciences, Catholic University of Valencia San Vicente Mártir, Valencia, Spain; 4 Veterinary and Experimental Science Faculty, Catholic University of Valencia San Vicente Mártir, Valencia, Spain

**Keywords:** biochemistry, hematology, lung ultrasound, ovine respiratory complex, post-mortem examination

## Abstract

**Introduction:**

Respiratory diseases are a major health concern in intensive lamb production, leading to significant economic losses and compromised animal welfare. This study aimed to characterize the hematological and biochemical profiles of lambs affected by ovine respiratory complex according to disease severity assessed by clinical, ultrasonographic, and post-mortem scoring systems.

**Methods:**

89 Lacaune lambs from a single farm were evaluated using a clinical respiratory score, lung ultrasound examination, and macroscopic post-mortem lung assessment, with severity classified into four categories, scores 0 to 3. Blood samples were collected for complete blood count and serum biochemistry analysis. Statistical comparisons were performed using one-way ANOVA and Tukey’s *post hoc* test.

**Results:**

Diseased lambs showed significant increases in WBC counts and NEU percentages, with concurrent lymphocytopenia and eosinophilia. RBC parameters varied with disease stage, showing anemia in moderate cases and compensatory increases in severe chronic cases. Biochemically affected lambs exhibited hypoglycemia, decreased ALP activity, hypoalbuminemia, hyperglobulinemia, and hypophosphatemia. Ultrasonographic scores demonstrated greater concordance with post-mortem findings and blood profile alterations compared to clinical scores.

**Discussion:**

These results indicate that combining lung ultrasound with hematological and biochemical analysis provide a more efficient evaluation of respiratory disease severity in lambs than a clinical evaluation.

## 1 Introduction

Respiratory diseases are a major health issue in farm and feedlot systems, leading to increased mortality, compromised animal welfare, and substantial economic losses (1–3). Among these, the ovine respiratory complex (ORC) constitutes the main respiratory syndrome affecting lambs; with a high prevalence, it impacts significantly their growth performance (length of the fattening period) and enhances both veterinary costs and mortality rates (4). The ORC is a multifactorial condition involving the interplay of infectious agents, the host’s immune response, and various environmental factors (5). Moreover, it has been demonstrated that viral agents such as respiratory syncytial virus and parainfluenza type 3 (PI3) increase the susceptibility to secondary infections, which are typically caused by *Mannheimia haemolytica*, *Pasteurella multocida*, *Mycoplasma* spp., and *Bibersteinia trehalosi* (5–7). Furthermore, various other bacterial species, such as *Trueperella pyogenes*, *Staphylococcus* spp., *Streptococcus* spp., and *Pseudomonas* spp., may occasionally contribute to respiratory infections in lambs (5, 8). Once the infectious dose threshold is reached, the host’s susceptibility and nonspecific defense mechanisms are compromised, allowing the disease to occur (9). Respiratory diseases frequently arise during critical stages of the lamb’s life cycle, with weaning representing a particularly susceptible period (5). It has been demonstrated that stress-inducing events, such as weaning, can lead to immune dysregulation, thereby increasing the risk of developing respiratory infections (10, 11). Animals with ORC usually exhibit nonspecific symptoms, including fever, anorexia, dyspnea, cough, and nasal and ocular discharge (12, 13). These symptoms can manifest in the entire herd or individual animal, and their severity can vary within the same animal. Additionally, there are three main clinical forms: hyperacute, acute, and chronic (5, 14). The clinical diagnosis of ORC is traditionally conducted through the evaluation of symptoms under field conditions. Recently, it has been demonstrated that this approach can be complemented by lung ultrasound (LUS), which enables the detection of lesion variations and allows for scoring based on the severity of ultrasonographic findings (15). In healthy lungs, lung ultrasound (LUS) typically reveals A-lines—horizontal reverberation artifacts parallel to the pleural line—caused by the reflection of ultrasound waves from air-filled alveoli (15–19). Moreover, LUS serves as a valuable diagnostic tool for identifying pathological pulmonary and pleural alterations in both young and adult animals. The most frequent ultrasonographic finding on LUS is the presence of non-aerated pulmonary areas consistent with consolidation, defined as the replacement of normally aerated lung tissue by inflammatory exudate (proteins, fibrin, inflammatory cells) or fibrous or necrotic tissue (3, 7, 16), and comet-tail artifacts (B-lines), which appear as vertical hyperechoic beams originating from the pleural line and extending through the pulmonary parenchyma (20). These findings were most frequently observed in the cranioventral lung lobes (15).

Hematological tests have been extensively utilized for the diagnosis of a multitude of diseases and the assessment of nutritional status in animals (21, 22). Information obtained from blood parameters would serve to corroborate the findings of the physical examination and, in conjunction with the clinical history, would provide an excellent basis for medical judgment (23). Respiratory infections in sheep have the potential to impact several hematological parameters, including leukocytes and erythrocytes (21). These alterations may indicate the severity of the infection and the extent of the inflammatory response, but they are not specific to a respiratory disease (24). Nevertheless, the distinction in lymphocyte levels before and following the experimental induction of *Pasteurella multocida* in goats, along with their correlation with respiratory infection, can be emphasized (25).

Blood biochemistry is an essential diagnostic tool, providing information on the metabolic, nutritional, and pathological status of animals (26, 27). The biochemical composition of blood includes plasma proteins, enzymes, electrolytes, metabolites, and hormones, offering an integrated assessment of the functional status of multiple organ systems (23). Plasma proteins represent approximately 6–8% of blood volume and play a key role in maintaining oncotic pressure, molecular transport, and immune function (27). Albumin, synthesized in the liver, is the main determinant of oncotic pressure and a major transport protein. The albumin-to-globulin ratio is a valuable indicator of inflammatory and nutritional status (28). During acute inflammatory responses, hepatic synthesis of positive acute-phase proteins increases, while albumin production decreases, resulting in characteristic alterations in serum protein electrophoretic profiles (29). Enzymatic markers provide complementary biochemical information for the assessment of tissue damage and organ function (27). In the context of respiratory disease, damage to the pulmonary epithelium may contribute to increased serum activities of these enzymes; however, the lack of tissue specificity and the overlap with isoenzymes from other organs limit their diagnostic specificity (30). Electrolyte balance and acid–base status represent crucial biochemical parameters that may be significantly altered during respiratory disease (31).

The aim of this study was to characterize the hematological and biochemical profiles of lambs affected by ORC according to disease severity, as assessed by clinical, ultrasonographic, and post-mortem scoring systems.

## 2 Materials and methods

### 2.1 Study design and animals

An observational diagnostic agreement study was conducted in April 2025 on an intensive dairy sheep farm located in Catadau, Valencian Community, Spain. A total of 89 Lacaune lambs were included in the study, comprising 64.29% (57/89) males and 35.71% (32/89) females. The farm operates as a free-stall facility with controlled feeding and reproduction programs to maintain continuous lactation throughout the year.

Immediately after birth, lambs were separated from their dams and transferred to artificial lactation facilities. During this phase, they were fed a commercial milk replacer (ELVOR 63: crude protein 24%, fat 24%, fiber 5%, ash 7%, calcium 0.9%, sodium 0.45%, phosphorus 0.75%), cereal straw, and commercial starter feed (Lactoiniciacor Nanta: crude protein 18%, crude fiber 4%, fat 3%, ash 6.9%, starch and sugars 43%). Milk replacer consumption gradually declined until weaning at approximately 40 days of age, at which point lambs weighed approximately 17 kg.

Following weaning, a pooled fecal sample was taken, and lambs were subjected to coprological analysis to rule out the presence of endoparasites and were subsequently transferred to the fattening facilities, where they remained until 70 days of age, reaching an average weight of 28 kg. During the fattening phase, lambs were housed in pens measuring 3 × 13 meters (approximately 40 animals per pen) and were provided with cereal straw and *ad libitum* access to concentrated fattening feed (Nantacor Intensive Fattening Nanta: crude protein 17.5%, fiber 4.3%, fat 3%, ash 42%, starch and sugars 42%).

Animals included in the study were those presenting disorders potentially associated with impaired growth or with locomotor, digestive, or respiratory symptoms. All animals remaining suitable for transport to the slaughterhouse, where samples were obtained for pathological examination.

### 2.2 Clinical respiratory scoring

Clinical evaluation was performed using an adapted version of the Wisconsin Calf Respiratory Score (WCRS) (15, 32). Five clinical parameters were systematically assessed: ocular discharge (OD), nasal discharge (ND), head tilting (HT), cough, and rectal temperature (RT). Each parameter was graded on a 4-point scale (0–3), where 0 indicated absence of clinical signs or normal physiological values, and 3 represented the most severe manifestation (Table 1).

**Table 1:** Clinical symptom evaluation for the diagnosis of ORC.

Diagnosis^a^	Score			
	0	1	2	3
OD	No	Mild unilateral serous discharge	Bilateral serous discharge	Bilateral mucosal discharge
ND	No	Mild unilateral serous discharge	Unilateral serous discharge	Bilateral mucosal discharge
HT	No	Head movement	Unilateral head drooping	Bilateral head drooping
COUGH	No	Induced and single cough	Induced and multiple or Spontaneous and occasional	Spontaneous and multiple
RT	< 39.49 °C	39.50 °C -39.89 °C	39.90 °C-40.49 °C	≥ 40.50 °C

^a^Score clinic parameters: OD, ocular discharge; ND, nasal discharge; HT, head tilting; and RT, rectal temperature.

The cumulative clinical score (SClinic) was calculated as the sum of individual parameter scores, yielding a total score ranging from 0 to 15. Based on this cumulative value, lambs were stratified into four severity categories: score 0 (cumulative value 0–2), score 1 (cumulative value 3–7), score 2 (cumulative value 8–11), and score 3 (cumulative value 12–15) (Table 2).

**Table 2:** Scoring system for clinical, ultrasonographic, and post-mortem lung evaluation in lambs.

Score	SClinic	SUlt^a^	SPost^b^
0	The resultant value, which ranges from 0 to 2, is the sum of the constituent parts that form the clinical score.	Normal lung	<10%
1	The resultant value, which ranges from 3 to 7, is the sum of the constituent parts that form the clinical score.	>5 B-lines, no CON	10–20%
2	The resultant value, which ranges from 8 to 11, is the sum of the constituent parts that form the clinical score.	>5 B-lines, <5 CON	20–30%
3	The resultant value, which ranges from 12 to 15, is the sum of the constituent parts that form the clinical score.	>5 CON, PF o ABS	>30%

^a^B-lines, comet tails; CON, consolidation; PF, pleural effusion; ABS, abscess.

^b^Spost represents the percentage of pulmonary areas affected by each animal in post-mortem examination.

### 2.3 Lung ultrasonography

Thoracic ultrasonography was performed using an Esaote MyLab One Vet ultrasound system (Esaote España, Barcelona, Spain) equipped with a micro-convex transducer (SC3123). The reference frequency was set at 10 MHz with an imaging depth of 8 cm. Hydroalcoholic gel was applied to the thoracic wall to optimize acoustic coupling and enhance image quality.

Examinations were conducted in a dimly lit environment to improve screen visualization. Each lamb was gently positioned in lateral recumbency on a padded surface, and the examination was performed sequentially, beginning with the left hemithorax, followed by the right. A systematic scanning protocol was employed to evaluate the cranial, middle, and caudal lung lobes at standardized intercostal spaces:

*Cranial lobe*: Probe positioned between the 2nd and 3rd intercostal spaces, with pulmonary arteries and veins serving as anatomical landmarks.

*Middle lobe (right)*: Ventral region assessed at the 4th–5th intercostal space above the sternum (heart as reference); dorsal region examined between the 5th and 6th intercostal spaces.

*Caudal lobe*: Ventral region scanned at the 7th–9th intercostal spaces (liver as reference); dorsal region evaluated between the 8th and 10th intercostal spaces (diaphragm as landmark).

Ultrasonographic findings were classified into five categories: A-lines (normal), B-lines (comet-tail artifacts), consolidation (CON), pleural effusion (PE), and abscess (ABS). Based on the type and distribution of lesions, lambs were assigned an ultrasound score (SUlt) ranging from 0 to 3: score 0 (normal lungs with predominant A-lines), score 1 (>5 sites with B-lines, no consolidation), score 2 (>5 sites with B-lines and <5 sites with consolidation), and score 3 (>5 sites with consolidation, pleural effusion, or abscess) (Table 2).

### 2.4 Blood sample collection and laboratory analysis

Blood samples were collected immediately following clinical and ultrasonographic examinations. Venipuncture was performed by trained personnel from the external jugular vein using a 0.8 × 25 mm beveled needle (Terumo Neolus, Belgium). Two sample types were obtained from each animal:

1. Hematological analysis: Blood was collected into EDTA-containing tubes (Vacuette® K3E EDTA, 2 mL) and analyzed within 24 h of collection using an automated hematology analyzer (Melet Schoering MS4s® HES233). The complete blood count included: white blood cell count (WBC) with five-part differential (lymphocytes [LYM], monocytes [MON], neutrophils [NEU], eosinophils [EOS], and basophils [BAS]); red blood cell parameters (RBC count, hemoglobin [HGB], hematocrit [HTO], mean corpuscular volume [MCV], mean cell hemoglobin [HbCM], mean cell hemoglobin concentration [CHbCM], and red cell distribution width [RDW]); and platelet parameters (platelet count [PLT], mean platelet volume [MPV], plateletcrit [PTC], and platelet distribution width [PDW]).
2. Biochemical analysis: Blood was collected into plain tubes, allowed to clot at room temperature, and centrifuged to obtain serum, which was stored at −20 °C until analysis. Biochemical parameters were measured using an automated analyzer (Metrolab 2,300, Wiener Lab., Rosario, Argentina) and included: metabolic markers (glucose [GLUC], cholesterol [CHOL], triglycerides [TGL]); protein profile (total protein [TP], albumin [ALB], globulins [GLOB]); renal function markers (urea, creatinine [CREA]); hepatic enzymes (alkaline phosphatase [ALP], alanine aminotransferase [ALT/GPT], aspartate aminotransferase [AST/GOT], gamma-glutamyl transferase [GGT], total bilirubin [TB]); electrolytes (calcium [Ca], phosphorus [P]); and muscle enzyme (creatine kinase [CK-NAC]).

All samples were maintained at 4 °C in a portable refrigerated container during transport to the laboratory.

### 2.5 Post-mortem examination

A standardized macroscopic post-mortem examination was performed at the abattoir approximately 12 h following the *in vivo* assessment. Lungs were systematically examined, and lesions were characterized according to anatomical distribution, delineation, morphological features, coloration, dimensions, texture, consistency, and overall extent of involvement.

Digital quantification of affected lung areas was performed using Adobe® Photoshop® CC2023 (version 24.0). The percentage of pulmonary consolidation was calculated for each animal, and pneumonia severity was classified using a standardized post-mortem score (SPost) ranging from 0 to 3: score 0 (<10% consolidation), score 1 (10–20% consolidation), score 2 (20–30% consolidation), and score 3 (>30% consolidation) (Table 2).

### 2.6 Statistical analysis

All statistical analyses were performed using IBM SPSS Statistics (version 29.0.2.0). Descriptive statistics, including means and standard errors (SE), were calculated for each hematological and biochemical parameter within each scoring category.

Normality of data distribution was assessed using the Shapiro–Wilk test. Comparisons of hematological and biochemical parameters among severity categories (scores 0–3) were performed using one-way analysis of variance (ANOVA). When significant differences were detected (*p* < 0.05), Tukey’s honestly significant difference (HSD) *post hoc* test was applied for pairwise comparisons to control for Type I error in multiple testing. Different superscript letters within rows indicate statistically significant differences between groups, whereas shared letters indicate no statistical difference. A significance level of *α* = 0.05 was used for all statistical tests.

## 3 Results

Distribution of lambs according to clinical (SClinic), ultrasonographic (SUlt), and post-mortem (SPost) lung evaluation of this study is shown in Table 3.

**Table 3:** Distribution of lambs according to clinical (SClinic), ultrasonographic (SUlt), and post-mortem (SPost) lung evaluation in lambs.

Score	SClinic	SUlt	SPost
0	34.83% (31/89)	26.96% (24/89)	35.95% (32/89)
1	35.95% (32/89)	21.34% (19/89)	20.22% (18/89)
2	21.34% (19/89)	24.71% (22/89)	20.22% (18/89)
3	7.86% (7/89)	26.96% (24/89)	23.59% (21/89)

### 3.1 Hematological results

The hematological profile was analyzed in relation to the SClinic results (Table 4). Significant differences among clinical groups were observed for WBC, LYM, NEU, EOS, MON, BAS, RBC, HTO, HGB, MCV, RDW, HbCM, and PDW (*p* < 0.05). In contrast, CHbCM, PLT, MPV, and PTC did not show significant differences among SClinic scores (*p* > 0.05).

**Table 4:** Results of the evaluation of the hematological profile in lambs according to their clinical score for ORC (mean ± SE).

Parameter	Clinical score				SL
	0	1	2	3	
WBC m/mm^3^	9.01 ± 0.72^a^	11.50 ± 1.40^b^	11.32 ± 2.15^bc^	16.67 ± 3.36^c^	<0.05
LYM %	46.16 ± 2.89^a^	31.17 ± 2.96^b^	29.39 ± 3.42^b^	36.87 ± 7.83^b^	<0.05
MON %	5.29 ± 0.38^a^	4.08 ± 0.26^b^	4.52 ± 0.50^ab^	2.91 ± 0.89^b^	<0.05
NEU %	45.47 ± 2.90^a^	58.11 ± 3.63^b^	62.34 ± 3.47^b^	57.43 ± 7.73^b^	<0.05
EOS %	1.59 ± 0.23^a^	6.16 ± 1.71^b^	3.39 ± 0.65^c^	2.51 ± 0.60^c^	<0.05
BAS %	1.60 ± 0.37^a^	0.68 ± 0.17^b^	0.47 ± 0.05^b^	0.25 ± 0.15^c^	<0.05
RBC m/mm^3^	12.37 ± 0.39^a^	11.26 ± 0.34^b^	10.78 ± 0.53^bc^	13.08 ± 2.21^c^	<0.05
MCV fL	27.09 ± 0.38^a^	26.40 ± 0.78^ab^	25.35 ± 0.86^b^	30.33 ± 2.01^c^	<0.05
HTO %	31.73 ± 1.37^ac^	29.22 ± 1.37^a^	27.14 ± 1.59^b^	35 ± 4.04^c^	<0.05
HbCM pg	9.32 ± 0.13^a^	8.75 ± 0.26^b^	9 ± 0.20^abc^	9.89 ± 0.72^ac^	<0.05
CHbCM g/dL	34.77 ± 0.72^a^	34.74 ± 1.94^a^	36.8 ± 2.02^a^	35.31 ± 1.82^a^	ns
RDW	18.11 ± 0.52^a^	16.20 ± 0.32^b^	15.62 ± 0.46^b^	17.60 ± 1.68^ab^	<0.05
HGB g/dL	11.39 ± 0.33^a^	10.04 ± 0.42^b^	9.93 ± 0.57^b^	11.38 ± 0.47^a^	<0.05
PLT m/mm^3^	521.5 ± 40.1^a^	460.7 ± 32.1^a^	473.9 ± 45.7^a^	465.2 ± 39.9^a^	ns
MPV fl	6.98 ± 0.18^a^	6.97 ± 0.09^a^	7.10 ± 0.11^a^	7.10 ± 0.21^a^	ns
PTC %	0.36 ± 0.03^a^	0.32 ± 0.02^a^	0.30 ± 0.03^a^	0.32 ± 0.02^a^	ns
PDW	25.26 ± 5.55^a^	9.76 ± 1.85^b^	7.59 ± 0.51^bc^	6.59 ± 0.83^c^	<0.05

The results include mean values, standard errors (SE), and significant levels (SL) among groups based on Tukey’s test. Different letters in the same row indicate significant differences between groups, whereas shared letters indicate no statistical difference.

Thus, according to the SClinic results and regarding leukocytic parameters, WBC counts were significantly higher in lambs with SClinic scores of 1 (11.50 ± 1.40), 2 (11.32 ± 2.15), and 3 (16.67 ± 3.36) compared to lambs with a score of 0. The LYM percentage decreased in all SClinic scores different from 0, with the lowest values observed in score 2 (29.39 ± 3.42), which differed significantly from score 0. Conversely, NEU percentage increased significantly with increasing clinical severity, reaching the highest values in scores 2 (62.34 ± 3.47) and 3 (57.43 ± 7.73). EOS was significantly higher in lambs with a SClinic of 1 (6.16 ± 1.71), whereas scores 2 and 3 showed significantly lower values compared to score 0. MON showed a significant decrease in lambs with a clinical score of 3 (2.91 ± 0.89) compared to the other groups. BAS progressively decreased with increasing clinical scores, reaching the lowest values in score 3. Regarding erythrocytic parameters, RBC count was significantly higher in lambs with a clinical score of 3 (13.08 ± 2.21) compared to scores 1 and 2, which showed a significant decrease. HTO was also elevated in lambs with a SClinic of 3 (35 ± 4.04) compared to lower scores. HGB followed a similar trend, with significant differences between groups 1 and 2 compared to groups 0 and 3. MCV was significantly higher in lambs with a clinical score of 3 (30.33 ± 2.01) compared to lower scores. RDW was highest in score 3 (17.60 ± 1.68), although it did not differ significantly among the remaining groups. Lambs with a score of 1 showed a significant decrease in HbCM, whereas those with a score of 3 exhibited a significant increase. PDW significantly decreased with increasing clinical severity, being lowest in scores 2 and 3.

The hematological profile analyzed according to SUlt results (Table 5) showed significant differences among ultrasound severity groups for WBC, LYM, NEU, EOS, BAS, RBC, HTO, HGB, MCV, RDW, PLT, MPV, and PDW (*p* < 0.05). In contrast, MON did not show significant differences among SUlt scores.

**Table 5:** Results of the evaluation of the hematological profile in lambs according to their ultrasound score for ORC (mean ± SE).

Parameter	Ultrasound score				SL
	0	1	2	3	
WBC m/mm^3^	8.90 ± 0.90^a^	10.94 ± 1.46^a^	9.26 ± 1.09^a^	15.93 ± 2.20^b^	<0.05
LYM %	51.95 ± 2.94^a^	30.21 ± 3.12^b^	32.72 ± 3.31^b^	29.34 ± 3.47^b^	<0.05
MON %	4.89 ± 0.38^a^	4.51 ± 0.36^a^	4.72 ± 0.40^a^	3.98 ± 0.53^a^	ns
NEU %	39.89 ± 2.88^a^	56.93 ± 4.63^b^	58.60 ± 3.42^b^	63.76 ± 3.48^b^	<0.05
EOS %	1.63 ± 0.21^a^	7.63 ± 2.77^b^	3.98 ± 0.82^c^	2.38 ± 0.36^c^	<0.05
BAS %	1.99 ± 0.45^a^	0.78 ± 0.28^b^	0.41 ± 0.05^c^	0.43 ± 0.05^c^	<0.05
RBC m/mm^3^	12.82 ± 0.45^a^	11.51 ± 0.39^ab^	10.69 ± 0.55^b^	11.60 ± 0.68^b^	<0.05
MCV fL	27.35 ± 0.38^a^	26.84 ± 0.72^ab^	25.34 ± 1.00^b^	27.27 ± 0.97^ab^	<0.05
HTO %	32.67 ± 1.63^a^	30.03 ± 1.69^abc^	26.96 ± 1.72^b^	30.48 ± 1.61^ac^	<0.05
HbCM pg	9.48 ± 0.15^a^	9.04 ± 0.16^b^	8.25 ± 0.32^c^	9.52 ± 0.27^a^	<0.05
CHbCM g/dL	34.92 ± 0.55^a^	34.33 ± 1.21^a^	34.86 ± 2.89^a^	36.62 ± 1.50^a^	ns
RDW	18.26 ± 0.56^a^	16.22 ± 0.45^a^	16.11 ± 0.55^a^	16.63 ± 0.62^a^	ns
HGB g/dL	11.93 ± 0.33^a^	10.45 ± 0.43^b^	9.20 ± 0.63^c^	10.63 ± 0.30^b^	<0.05
PLT m/mm^3^	569.5 ± 47.6^a^	406.3 ± 36.5^b^	461.3 ± 45.6^bc^	484.7 ± 26.7^c^	<0.05
MPV fl	6.92 ± 0.23^ab^	6.89 ± 0.08^a^	7.13 ± 0.13^b^	7.01 ± 0.98^ab^	<0.05
PTC %	0.39 ± 0.37^a^	0.28 ± 0.03^a^	0.23 ± 0.42^a^	0.33 ± 0.02^a^	ns
PDW	29.66 ± 6.66^a^	11.19 ± 3.05^b^	7.27 ± 0.52^b^	7.60 ± 0.35^b^	<0.05

The results include mean values, standard errors (SE), and significant levels (SL) among groups based on Tukey’s test. Different letters in the same row indicate significant differences between groups, whereas shared letters indicate no statistical difference. Ns, not significant.

Thus, according to the SUlt results and regarding leukocytic parameters, lambs with a SUlt of 3 exhibited higher WBC counts (15.93 ± 2.20) compared to lower scores. The LYM percentage decreased with increasing ultrasound severity, with scores 1–3 showing lower values than score 0. Conversely, NEU percentage increased progressively, reaching its highest value in score 3 (63.76 ± 3.48). EOS showed a distinct pattern, being higher in score 1 (7.63 ± 2.77) compared to the remaining groups. BAS decreased significantly with increasing ultrasound scores, with the lowest values observed in scores 2 and 3. MON remained relatively stable across SUlt categories. Regarding erythrocytic parameters, RBC count was lower in lambs with a SUlt of 2 (10.69 ± 0.55) compared to the other groups. HTO was reduced in lambs with a SUlt of 2 (26.96 ± 1.72) compared to scores 0 and 3. HGB also showed differences, with the lowest values observed in scores 2 and 3. MCV was lower in lambs with a SUlt of 2 (25.34 ± 1.00) compared to the remaining groups. RDW was reduced in scores 1–3 compared to score 0. For platelet-related parameters, PLT was lower in lambs with a SUlt of 1 (406.3 ± 36.5) compared to scores 0, 2, and 3. MPV was higher in lambs with SUlt scores of 2 and 3 (7.13 ± 0.13 and 7.01 ± 0.98, respectively) compared to lower scores. PDW decreased progressively with increasing SUlt severity, with the lowest values recorded in scores 2 and 3.

Finally, according to the SPost (Table 6), the hematological profile showed significant differences among severity groups observed for WBC, LYM, NEU, EOS, BAS, RBC, MCV, HTO, HGB, RDW, PLT, and PDW (*p* < 0.05). In contrast, MON, HbCM, CHbCM, MPV, and PTC did not show significant differences among post-mortem scores.

**Table 6:** Results of the evaluation of the hematological profile in lambs according to their macroscopic post-mortem examination score for ORC (mean ± SE).

Parameter	Post-mortem score				SL
	0	1	2	3	
WBC m/mm^3^	10.14 ± 1.32^a^	10.26 ± 1.34^a^	10.75 ± 1.56^a^	14.52 ± 1.96^b^	<0.05
LYM %	44.66 ± 3.28^a^	35.03 ± 3.34^b^	29.38 ± 3.36^b^	29.75 ± 3.68^b^	<0.05
MON %	4.79 ± 0.28^a^	4.84 ± 0.49^a^	4.21 ± 0.40^a^	4.04 ± 0.60^a^	ns
NEU %	46.44 ± 3.30^a^	53.61 ± 4.39^ab^	60.50 ± 3.93^bc^	62.79 ± 3.79^c^	<0.05
EOS %	1.76 ± 0.25^a^	5.76 ± 2.49^bc^	6.02 ± 1.92^b^	2.88 ± 0.40^c^	<0.05
BAS %	1.58 ± 0.36^a^	0.83 ± 0.29^b^	0.44 ± 0.06^c^	0.42 ± 0.06^c^	<0.05
RBC m/mm^3^	12.16 ± 0.45^a^	11.44 ± 0.41^ab^	10.74 ± 0.51^b^	12.00. ± 0.78^ab^	<0.05
MCV fL	26.43 ± 0.67^a^	25.31 ± 1.14^a^	26.78 ± 0.40^a^	28.34 ± 0.88^b^	<0.05
HTO %	30.36 ± 1.57^ab^	27.99 ± 1.93^a^	28.89 ± 1.43^a^	32.58 ± 1.68^b^	<0.05
HbCM pg	9.06 ± 0.24^a^	9.02 ± 0.16^a^	8.78 ± 0.23^a^	9.10 ± 0.13^a^	ns
CHbCM g/dL	35.59 ± 1.82^ab^	37.46 ± 2.32^a^	32.98 ± 0.80^b^	34.74 ± 1.10^ab^	<0.05
RDW	17.95 ± 0.51^a^	15.79 ± 0.55^b^	15.80 ± 0.40^b^	17.04 ± 0.29^c^	<0.05
HGB g/dL	11.03 ± 0.46^a^	10.46 ± 0.44^ab^	9.60 ± 0.55^b^	10.91 ± 0.37^ab^	<0.05
PLT m/mm^3^	550.0 ± 41.15^a^	459.56 ± 43.87^b^	439.76 ± 44.01^b^	446.91 ± 26.59^b^	<0.05
MPV fl	7.02 ± 0.19^a^	7.09 ± 0.14^a^	6.89 ± 0.09^a^	7.05 ± 0.09^a^	ns
PTC %	0.38 ± 0.03^a^	0.29 ± 0.03^b^	0.33 ± 0.04^ab^	0.32 ± 0.02^ab^	<0.05
PDW	24.61 ± 5.41^a^	11.53 ± 3.68^b^	7.72 ± 0.52^b^	7.58 ± 0.39^b^	<0.05

The results include mean values, standard errors (SE), and significant levels (SL) among groups based on Tukey’s test. Different letters in the same row indicate significant differences between groups, whereas shared letters indicate no statistical difference. Ns, not significant.

According to SPost results and regarding leukocytic parameters, WBC counts were higher in lambs with a score of 3 (14.52 ± 1.96) compared to scores 0–2. The LYM percentage decreased significantly in scores 1–3, with values ranging from approximately 29–35%, compared to score 0 (44.66 ± 3.28). Conversely, NEU percentage increased progressively with lesion severity, reaching 62.79 ± 3.79 in score 3. EOS showed a distinct pattern, being higher in scores 1 (5.76 ± 2.49) and 2 (6.02 ± 1.92), whereas score 3 exhibited a marked decrease (2.88 ± 0.40). BAS decreased progressively with increasing scores, reaching the lowest values in scores 2 and 3. MON remained stable across groups. Regarding erythrocytic parameters, RBC count was lower in lambs with a score of 2 (10.74 ± 0.51) compared to score 0 (12.16 ± 0.45). MCV was higher in score 3 (28.34 ± 0.88) compared to the remaining groups, and HTO was also elevated in score 3 (32.58 ± 1.68). HGB was lower in score 2, while intermediate values were observed in the other groups. RDW was highest in score 0 and lower in scores 1 and 2, with score 3 showing intermediate values. No differences were detected for HbCM or CHbCM, although CHbCM showed slightly lower values in score 2. In terms of platelet parameters, PLT and PDW were lower in scores 1–3 compared to score 0, with PDW showing a marked reduction in score 3. MPV and PTC did not differ among groups.

### 3.2 Biochemical results

The biochemical profile analyzed in relation to the SClinic results (Table 7) showed significant differences among clinical groups for GLUC, CHOL, TGL, TP, ALB, GLOB, CREA, ALP, ALT, AST, TB, Ca, P, and CK-NAC (*p* < 0.05). In contrast, UREA and GGT did not show significant differences among clinical scores.

**Table 7:** Results of the evaluation of the biochemical profile in lambs according to their clinical examination score for ORC (mean ± SE).

Parameter	Clinical score				SL
	0	1	2	3	
GLUC mg/dL	59.40 ± 4.13^a^	34.86 ± 6.28^b^	32.68 ± 5.48^bc^	18.35 ± 9.72^c^	<0.05
CHOL (mg/dL)	49.12 ± 5.34^b^	75.60 ± 10.22^a^	62.48 ± 9.05^ab^	47.15 ± 13.48^b^	<0.05
TGL (mg/ml)	22.95 ± 2.52^a^	21.18 ± 2.47^a^	17.94 ± 3.57^ab^	14.50 ± 2.88^b^	<0.05
TP (g/dL)	6.53 ± 0.23^b^	7.51 ± 0.21^a^	6.70 ± 0.42^b^	6.81 ± 0.31^b^	<0.05
ALB g/dL	3.16 ± 0.09^a^	2.96 ± 0.75^ab^	2.82 ± 0.14^b^	2.85 ± 0.11^ab^	<0.05
GLOB g/dL	3.34 ± 0.19^b^	4.54 ± 0.24^a^	4.01 ± 0.33^a^	3.98 ± 0.29^a^	<0.05
UREA	39.10 ± 3.44^a^	41.68 ± 3.71^a^	43.62 ± 9.31^a^	43.76 ± 4.20^a^	ns
CREA mg/dL	0.63 ± 0.26^ab^	0.68 ± 0.05^a^	0.79 ± 0.20^a^	0.49 ± 0.07^b^	<0.05
ALP UI/I	447.90 ± 66.45^a^	198.07 ± 26.34^b^	171.06 ± 28.77^b^	210 ± 68.08^b^	<0.05
ALT/GPT UI/I	11.53 ± 1.46^a^	7.54 ± 0.85^b^	7.94 ± 1.99^b^	5.83 ± 1.30^b^	<0.05
AST/GOT UI/I	100.40 ± 6.05^ab^	86.87 ± 6.80^b^	93.70 ± 15.62^ab^	148.93 ± 48.14^a^	<0.05
GGT UI/I	93.83 ± 16.81^a^	102.91 ± 19.75^a^	78.11 ± 8.88^a^	79.68 ± 11.36^a^	ns
TB mg/dL	0.08 ± 0.01^b^	0.18 ± 0.03^a^	0.24 ± 0.07^a^	0.17 ± 0.03^a^	<0.05
Ca mg/dL	9.27 ± 0.30^a^	9.18 ± 0.15^a^	8.68 ± 0.54^ab^	8.20 ± 0.28^b^	<0.05
P mg/dL	8.97 ± 0.45^a^	8.05 ± 0.32^b^	7.12 ± 0.56^c^	5.50 ± 0.54^d^	<0.05
CK-NAC UI/I	374.71 ± 29.97^b^	458.22 ± 44.03^a^	367.50 ± 71.19^a^	389.75 ± 281.08^a^	<0.05

The results include mean values, standard errors (SE), and significant levels (SL) among groups based on Tukey’s test. Different letters in the same row indicate significant differences between groups, whereas shared letters indicate no statistical difference. Ns: not significant.

According to SClinic results, GLUC concentrations were highest in score 0 (59.40 ± 4.13a), decreased in score 1 (34.86 ± 6.28b) and score 2 (32.68 ± 5.48bc), and reached the lowest values in score 3 (18.35 ± 9.72c). CHOL was higher in score 1 (75.60 ± 10.22a) compared to scores 0 and 3 (49.12 ± 5.34b and 47.15 ± 13.48b), while score 2 showed intermediate values (62.48 ± 9.05ab). TGL decreased, with score 3 (14.50 ± 2.88b) differing from score 0 (22.95 ± 2.52a). TP was higher in score 1 (7.51 ± 0.21a) compared to the remaining groups. ALB was higher in score 0 (3.16 ± 0.09a) compared to score 2 (2.82 ± 0.14b). GLOB increased in score 1 (4.54 ± 0.24a) compared to score 0 (3.34 ± 0.19b). CREA was lower in score 3 (0.49 ± 0.07b) compared to scores 1 and 2. ALP was markedly higher in score 0 (447.90 ± 66.45a) compared to scores 1–3. ALT was higher in score 0 (11.53 ± 1.46a) compared to scores 1–3. AST was highest in score 3 (148.93 ± 48.14a), differing from score 1. TB increased in scores 1–3 compared to score 0. Ca decreased progressively, with score 3 (8.20 ± 0.28b) differing from score 0 (9.27 ± 0.30a). P showed a clear stepwise decrease from score 0 (8.97 ± 0.45a) to score 3 (5.50 ± 0.54d). CK-NAC was higher in score 1 (458.22 ± 44.03a) compared to score 0 (374.71 ± 29.97b) (Table 7).

The biochemical profile evaluated according to SUlt results (Table 8) revealed significant differences for GLUC, TGL, TP, ALB, GLOB, UREA, ALP, ALT, AST, GGT, TB, Ca, and P (*p* < 0.05), whereas CHOL, CREA, and CK-NAC did not differ among ultrasound severity groups.

**Table 8:** Results of the evaluation of the biochemical profile in lambs according to their ultrasonographic examination score for ORC (mean ± SE).

Parameter	Ultrasound score				SL
	0	1	2	3	
GLUC mg/dL	61.91 ± 5.11^a^	51.27 ± 8.10^a^	32.23 ± 4.29^b^	22.67 ± 5.11^b^	<0.05
CHOL (mg/dL)	58.50 ± 5.46^a^	65.13 ± 13.71^a^	68.02 ± 11.33^a^	55.06 ± 8.67^a^	ns
TGL (mg/ml)	24.99 ± 3.01^a^	18.75 ± 3.69^ab^	19.47 ± 3.19^ab^	18.44 ± 2.19^b^	<0.05
TP (g/dL)	6.54 ± 0.27^b^	7.09 ± 0.26^a^	7.09 ± 0.35^ab^	7.07 ± 0.32^ab^	<0.05
ALB g/dL	3.26 ± 0.08^a^	3.05 ± 0.10^b^	2.90 ± 0.08^bc^	2.75 ± 0.11^c^	<0.05
GLOB g/dL	3.28 ± 0.23^b^	3.98 ± 0.19^b^	4.16 ± 0.37^ab^	4.42 ± 0.27^a^	<0.05
UREA	44.60 ± 3.91^a^	34.83 ± 3.58^b^	42.21 ± 9.20^ab^	41.85 ± 4.19^ab^	<0.05
CREA mg/dL	0.62 ± 0.02^a^	0.71 ± 0.07^a^	0.79 ± 0.20^a^	0.60 ± 0.04^a^	ns
ALP UI/I	479.87 ± 78.76^a^	250.31 ± 46.06^b^	197.71 ± 36.32^b^	178.35 ± 27.21^b^	<0.05
ALT/GPT UI/I	12.34 ± 1.58^a^	10.10 ± 1.46^ab^	7.05 ± 1.51^bc^	6.20 ± 1.25^c^	<0.05
AST/GOT UI/I	106.76 ± 6.39^a^	88.88 ± 9.81^b^	86.58 ± 13.17^b^	104.08 ± 15.42^ab^	<0.05
GGT UI/I	103.70 ± 20.86^a^	110.40 ± 33.35^ab^	82.96 ± 8.12^ab^	75.81 ± 5.52^b^	<0.05
TB mg/dL	0.09 ± 0.01^b^	0.14 ± 0.03^a^	0.21 ± 0.07^a^	0.19 ± 0.03^a^	<0.05
Ca mg/dL	9.55 ± 0.33^a^	8.98 ± 0.33^ab^	8.9 ± 0.27^b^	8.63 ± 0.37^b^	<0.05
P mg/dL	9.52 ± 0.54^a^	7.78 ± 0.43^b^	7.80 ± 0.36^b^	6.77 ± 0.43^b^	<0.05
CK-NAC UI/I	438.95 ± 31.54^a^	446.06 ± 65.50^a^	372.55 ± 52.21^a^	359.05 ± 84.02^a^	ns

The results include mean values, standard errors (SE), and significant levels (SL) among groups based on Tukey’s test. Different letters in the same row indicate significant differences between groups, whereas shared letters indicate no statistical difference. Ns, not significant.

According to SUlt results, GLUC was higher in scores 0 and 1 compared to scores 2 and 3. TGL was lower in score 3 compared to score 0. TP was higher in score 1 compared to score 0. ALB decreased progressively from score 0 to score 3. GLOB was highest in score 3, differing from score 0. UREA was lower in score 1 compared to score 0. ALP was highest in score 0 and reduced in scores 1–3. ALT decreased progressively, with score 3 differing from score 0. AST was lower in scores 1 and 2 compared to score 0. GGT decreased in score 3 compared to score 0. TB increased in scores 1–3 compared to score 0. Ca decreased from score 0 to scores 2 and 3. P was highest in score 0 and lower in scores 1–3 (Table 8).

The biochemical profile analyzed according to SPost results (Table 9) showed significant differences for GLUC, CHOL, TGL, TP, ALB, GLOB, UREA, ALP, ALT, AST, GGT, TB, Ca, and P (*p* < 0.05), whereas CREA and CK-NAC did not show significant variation among groups.

**Table 9:** Results of the evaluation of the biochemical profile in lambs according to their post-mortem examination score for ORC (mean ± SE).

Parameter	Post-mortem score				SL
	0	1	2	3	
GLUC mg/dL	56.30 ± 4.93^a^	36.21 ± 5.56^b^	35.43 ± 4.29^b^	29.54 ± 8.89^b^	<0.05
CHOL (mg/dL)	58.63 ± 6.78^b^	46.21 ± 10.25^b^	88.82 ± 13.38^a^	54.66 ± 7.45^b^	<0.05
TGL (mg/ml)	23.30 ± 2.52^a^	14.12 ± 1.63^b^	19.46 ± 3.94^ab^	22.95 ± 3.25^a^	<0.05
TP (g/dL)	6.72 ± 0.29^b^	7.32 ± 0.27^a^	6.73 ± 0.44^ab^	7.06 ± 0.19^ab^	<0.05
ALB g/dL	3.16 ± 0.08^a^	2.90 ± 0.09^b^	2.94 ± 0.17^b^	2.86 ± 0.08^b^	<0.05
GLOB g/dL	3.57 ± 0.27^b^	4.29 ± 0.26^a^	3.81 ± 0.33^ab^	4.32 ± 0.23^a^	<0.05
UREA	42.50 ± 3.25^a^	35.18 ± 3.10^b^	43.18 ± 10.48^ab^	43.18 ± 5.12^ab^	<0.05
CREA mg/dL	0.63 ± 0.02^a^	0.65 ± 0.05^a^	0.77 ± 0.23^a^	0.67 ± 0.07^a^	ns
ALP UI/I	447.59 ± 65.62^a^	236.31 ± 47.34^b^	141.33 ± 19.86^c^	190.74 ± 27.84^b^	<0.05
ALT/GPT UI/I	11.74 ± 1.47^a^	10.81 ± 1.84^a^	6.20 ± 1.01^b^	5.34 ± 0.89^b^	<0.05
AST/GOT UI/I	104.53 ± 6.58^a^	104.20 ± 14.54^a^	67.59 ± 6.16^b^	106.89 ± 17.63^a^	<0.05
GGT UI/I	96.61 ± 16.73^ab^	118.10 ± 33.88^a^	73.63 ± 5.68^b^	79.46 ± 4.59^ab^	<0.05
TB mg/dL	0.10 ± 0.02^b^	0.13 ± 0.02^ab^	0.22 ± 0.08^a^	0.21 ± 0.03^a^	<0.05
Ca mg/dL	9.39 ± 0.28^a^	9.13 ± 0.33^ab^	8.66 ± 0.58^ab^	8.70 ± 0.16^b^	<0.05
P mg/dL	9.08 ± 0.46^a^	7.25 ± 0.36^b^	7.76 ± 0.61^b^	7.16 ± 0.45^b^	<0.05
CK-NAC UI/I	438.12 ± 43.92^a^	415.48 ± 51.94^a^	425.56 ± 65.59^a^	320.42 ± 88.42^a^	ns

The results include mean values, standard errors (SE), and significant levels (SL) among groups based on Tukey’s test. Different letters in the same row indicate significant differences between groups, whereas shared letters indicate no statistical difference. Ns, not significant.

According to SPost results, GLUC was higher in score 0 compared to scores 1–3. CHOL was highest in score 2, differing from the remaining groups. TGL was lower in score 1 compared to scores 0 and 3. TP was higher in score 1 compared to score 0. ALB was higher in score 0 compared to scores 1–3. GLOB was higher in scores 1 and 3 compared to score 0. UREA was lower in score 1 compared to score 0. ALP was highest in score 0 and lowest in score 2. ALT was higher in scores 0 and 1 compared to scores 2 and 3. AST was lower in score 2 compared to the other groups. GGT was highest in score 1 and lowest in score 2. TB increased in scores 2 and 3 compared to score 0. Ca was higher in score 0 compared to score 3. P was higher in score 0 compared to scores 1–3 (Table 9).

## 4 Discussion

### 4.1 Overview of findings

This study provides a comprehensive characterization of the hematological and biochemical profiles associated with ORC in lambs, evaluated through three complementary diagnostic approaches: clinical scoring, lung ultrasonography, and post-mortem examination. Our findings demonstrate that disease severity, regardless of the assessment method employed, is consistently associated with significant alterations in both hematological and biochemical parameters. Importantly, the ultrasonographic scoring system showed greater concordance with post-mortem findings and tended to reflect stronger associations with blood profile alterations compared to clinical scoring alone.

Hematological and biochemical analyses have become integral components of the diagnostic approach to respiratory diseases in sheep (2, 26, 33, 34), providing valuable insights when clinical signs are nonspecific and serving as prognostic indicators (7). The growing application of automated analyzers for small ruminant blood samples has significantly expanded the utility of these diagnostic techniques (2).

### 4.2 Hematological alterations

#### 4.2.1 Leukocyte response

The leukocyte alterations observed in this study are characteristic of systemic inflammatory responses to respiratory infections. According to our results, SUlt and SPost appeared to accurately reflect the distinctions between lower and higher severity categories. While Sclinic showed greater overlap between intermediate scores. The significant leukocytosis and neutrophilia observed in lambs with higher severity scores across all three classification systems align with fundamental immunopathological principles outlined by Jain (35), who established that these changes represent hallmark features of acute bacterial infections. Similar patterns have been reported in calves with bovine respiratory disease (BRD) (30, 36), Buffaloes (18), sheep with pneumonia (33, 37), and lambs with Peste des Petits Ruminants (PPR) (38).

Higher neutrophil counts were generally associated with increasing severity scores and were more consistently observed when animals were classified using SUlt and SPost, whereas SClinic showed a more limited ability to discriminate between adjacent severity levels. The mechanisms underlying these changes likely involve bacterial stimulation of growth factors, pro-inflammatory cytokines, particularly IL-1, IL-6, and TNF-*α*, and other inflammatory mediators that promote leukocyte production, maturation, and release from bone marrow reserves (37, 39). Additionally, the observed association between neutrophilia and febrile episodes (38) suggests that fever may act as an additional stimulus for granulopoiesis in the context of ORC.

Lymphocyte counts tended to decrease as disease severity increased, particularly in animals classified within higher SUlt and Spost. This pattern was less clearly defined when clinical scoring was applied. The lymphocytopenia observed across all scoring systems is consistent with previous reports in pneumonic sheep (33) and calves with *Mycoplasma bovis* pneumonia (40). This phenomenon reflects the stress-induced release of endogenous glucocorticoids, which promote redistribution of circulating lymphocytes to lymphoid tissues (28, 33, 41, 42). Additionally, lymphocyte apoptosis secondary to overwhelming infection and type-1 hypersensitivity reactions involving histamine release may contribute to the observed lymphocytopenia (33, 43).

The significant eosinophilia observed in score 1 animals, with subsequent decline in more severely affected lambs, represents an interesting biphasic response. Initial eosinophil recruitment may reflect hypersensitivity reactions to microbial antigens (33, 43), while the subsequent decrease in severe cases could indicate eosinophil sequestration at inflammatory sites or bone marrow exhaustion during sustained inflammatory responses.

#### 4.2.2 Erythrocyte response

Erythrocyte-related alterations showed a severity-dependent pattern, likely reflecting different underlying pathophysiological mechanisms across disease stages. Subtle differences between severity scores were readily identified when animals were classified using SUlt and SPost, whereas SClinic showed a more limited ability to discriminate between severity categories. Lambs with mild to moderate disease (scores 1–2) exhibited significant decreases in RBC count, hemoglobin, and hematocrit, consistent with findings in pneumonic sheep (33, 37) and lambs with PPR (38). This anemia of chronic disease results from multiple mechanisms, including iron sequestration in macrophages mediated by hepcidin, direct suppression of erythropoiesis by inflammatory cytokines (IL-1, IL-6, TNF-*α*), and reduced erythrocyte survival due to microbial toxins and inflammatory mediators (44, 45).

In contrast, lambs with the highest severity scores (SClinic 3) showed paradoxically elevated RBC counts and hematocrit values. This finding, similar to observations in calves with chronic BRD (30), likely represents compensatory erythropoiesis in response to chronic hypoxemia. Animals with severe, chronic ORC experience sustained respiratory compromise that stimulates renal erythropoietin production, driving increased red blood cell production to maintain adequate tissue oxygenation. Additionally, dehydration associated with anorexia and increased respiratory water losses may contribute to hemoconcentration in severely affected animals (10, 46).

#### 4.2.3 Platelet parameters

Regarding platelet parameters, SUlt and SPost tended to identify lower platelet counts in animals classified with higher disease severity, whereas SClinic showed substantial overlap between severity categories. The thrombocytopenia and alterations in platelet indices observed in diseased lambs are consistent with findings reported in lambs affected by peste des petits ruminants (38) and likely reflect complex interactions among inflammation, coagulation processes, and platelet consumption. Under severe inflammatory conditions, platelets may be progressively depleted through activation of the coagulation cascade, adhesion to damaged endothelium, and sequestration within the pulmonary microvasculature (2, 28). In advanced cases of ovine respiratory complex (ORC) progressing toward endotoxemia or septicemia, the development of disseminated intravascular coagulation (DIC) may further contribute to platelet depletion.

### 4.3 Biochemical alterations

#### 4.3.1 Energy metabolism

The progressive hypoglycemia observed with increasing disease severity, particularly in animals classified with higher ultrasonographic and post-mortem scores, constitutes one of the most consistent and clinically relevant findings of this study. Glucose concentrations declined significantly from healthy controls to severely affected animals across all classification systems. This metabolic derangement results from the convergent effects of increased glucose utilization by activated immune cells and tissues undergoing repair, hepatic metabolic reprioritization during the acute phase response, and decreased feed intake secondary to anorexia and respiratory distress (30, 47–49). Similar findings have been reported in calves with BRD (30, 50) and goats with pneumonia (25), underscoring the metabolic burden imposed by respiratory infections.

#### 4.3.2 Protein metabolism

The alterations in serum protein profiles observed in this study reflect the hepatic acute phase response to inflammation. The significant hyperglobulinemia, coupled with hypoalbuminemia, particularly evident in SUlt and SPost classifications, represents the characteristic reprioritization of hepatic protein synthesis during inflammatory states (29, 51). During the acute phase response, the liver increases production of positive acute phase proteins (including immunoglobulins, fibrinogen, and C-reactive protein) while concurrently decreasing albumin synthesis. Additionally, increased capillary permeability associated with systemic inflammation promotes albumin extravasation into tissues and inflammatory exudates (8, 52). These protein profile alterations have been consistently reported in sheep with respiratory disease (33, 37) and calves with bronchopneumonia (30, 50).

#### 4.3.3 Enzyme activity

Enzyme activity displayed similar behavior across severity scores. Increases or decreases in enzyme activity were more evident in animals classified within higher SUlt and SPost categories, whereas SClinic showed weaker associations.

The marked decrease in ALP activity across all diseased groups represents a notable finding with important pathophysiological implications. In young growing animals, serum ALP predominantly originates from osteoblasts and reflects active bone formation during skeletal development (27, 53). The reduction in ALP observed in lambs with ORC suggests impaired bone metabolism secondary to the catabolic state induced by chronic infection, reduced nutrient intake, and systemic inflammation (54). These findings align with reports by Arbaga et al. (37), who attributed decreased ALP activity in pneumonic sheep to the negative metabolic impact of respiratory disease on growth and development.

The elevated AST activity observed in severely affected animals (SClinic 3) may indicate secondary hepatic involvement or increased muscle protein catabolism during advanced disease states (30, 55). Similar elevations have been documented in calves with bronchopneumonia (13, 50) and goats with mycoplasma pneumonia (44), suggesting that severe respiratory infections can compromise hepatic function through hypoxemia, endotoxemia, or direct inflammatory injury.

#### 4.3.4 Mineral metabolism

The progressive hypophosphatemia observed with increasing disease severity reflects multiple contributing factors, including reduced dietary intake, stress-induced alterations in renal phosphate handling, and inflammatory cytokine-mediated changes in mineral homeostasis (31, 56). Phosphorus plays crucial roles in energy metabolism (as a component of ATP), cellular signaling, and skeletal mineralization. Thus, persistent hypophosphatemia may contribute to muscle weakness, reduced growth performance, and prolonged recovery observed in lambs with ORC.

### 4.4 Comparative assessment of diagnostic approaches

A central finding of this study is the superior concordance between ultrasonographic and post-mortem scoring systems compared to clinical scoring. The hematological and biochemical profiles associated with SUlt showed remarkable similarity to those associated with SPost, with parallel patterns observed for leukocytosis, lymphocytopenia, neutrophilia, anemia, hypoglycemia, decreased ALP activity, hypoalbuminemia, hyperglobulinemia, and hypophosphatemia.

The distribution of lambs across severity categories further illustrates this diagnostic concordance. While SPost classified 23.59% of animals as score 3, SUlt identified a similar proportion (26.96%) with severe disease. In contrast, SClinic assigned a score of 3 to only 7.86% of lambs, with most animals concentrated in lower severity categories (scores 0–1: 70.78%). This observation suggests that clinical scoring alone may substantially underestimate true disease severity, consistent with findings in cattle, where Buczinski et al. (57) reported that LUS detected lung lesions in 29% of clinically healthy animals.

These findings highlight the diagnostic superiority of lung ultrasonography over clinical scoring for assessing respiratory disease severity in live animals. Previous studies demonstrated strong correlations (*r* = 0.92) between thoracic ultrasound findings and gross pulmonary lesions at necropsy (58), and LUS showed sensitivity and specificity approaching 90% for BRD detection compared to clinical symptom-based methods (59).

### 4.5 Clinical implications

The results of this study have several important clinical implications for the management of respiratory disease in intensive lamb production:

1. Enhanced diagnostic accuracy: The combination of lung ultrasonography with hematological and biochemical analysis provides a more comprehensive and accurate assessment of disease severity than clinical evaluation alone. This integrated approach enables identification of subclinically affected animals that may benefit from early intervention.
2. Prognostic value: The severity-dependent alterations in blood parameters may serve as prognostic indicators, helping clinicians identify animals at higher risk of complications or poor outcomes.
3. Treatment monitoring: Serial assessment of hematological and biochemical parameters could facilitate monitoring of treatment response and guide therapeutic decisions, including antibiotic selection and duration of therapy.
4. Reduced antibiotic use: Early and accurate diagnosis through LUS and blood analysis may enable more targeted antimicrobial therapy, contributing to antimicrobial stewardship efforts in sheep production (60).

## 5 Conclusion

This study demonstrates that hematological and biochemical profiles vary significantly according to the severity of ovine respiratory complex in lambs, as assessed by clinical, ultrasonographic, and post-mortem scoring systems. Diseased lambs exhibited leukocytosis, neutrophilia, lymphocytopenia, and alterations in erythrocyte parameters, along with hypoglycemia, decreased alkaline phosphatase activity, hypoalbuminemia, hyperglobulinemia, and hypophosphatemia. These changes reflect the systemic inflammatory response and metabolic alterations associated with respiratory infection. Notably, the hematological and biochemical profiles associated with ultrasonographic scores showed greater concordance with post-mortem findings compared to clinical scores alone, suggesting that lung ultrasound provides a more accurate assessment of true pulmonary lesion severity in live animals. The integration of lung ultrasound with hematological and biochemical analysis offers a comprehensive, non-invasive approach for the early detection and severity assessment of respiratory disease in lambs, potentially improving diagnostic accuracy, treatment decisions, and animal welfare outcomes in intensive lamb production systems.

## Data Availability

The raw data supporting the conclusions of this article will be made available by the authors, without undue reservation.
